# Exploring the matrix: knowledge, perceptions and prospects of artificial intelligence and machine learning in Nigerian healthcare

**DOI:** 10.3389/frai.2023.1293297

**Published:** 2024-01-19

**Authors:** Obi Peter Adigwe, Godspower Onavbavba, Saheed Ekundayo Sanyaolu

**Affiliations:** National Institute for Pharmaceutical Research and Development, Abuja, Nigeria

**Keywords:** artificial intelligence (AI), machine learning (ML), algorithms, clinical decision-making, healthcare

## Abstract

**Background:**

Artificial intelligence technology can be applied in several aspects of healthcare delivery and its integration into the Nigerian healthcare value chain is expected to bring about new opportunities. This study aimed at assessing the knowledge and perception of healthcare professionals in Nigeria regarding the application of artificial intelligence and machine learning in the health sector.

**Methods:**

A cross-sectional study was undertaken amongst healthcare professionals in Nigeria with the use of a questionnaire. Data were collected across the six geopolitical zones in the Country using a stratified multistage sampling method. Descriptive and inferential statistical analyses were undertaken for the data obtained.

**Results:**

Female participants (55.7%) were slightly higher in proportion compared to the male respondents (44.3%). Pharmacists accounted for 27.7% of the participants, and this was closely followed by medical doctors (24.5%) and nurses (19.3%). The majority of the respondents (57.2%) reported good knowledge regarding artificial intelligence and machine learning, about a third of the participants (32.2%) were of average knowledge, and 10.6% of the sample had poor knowledge. More than half of the respondents (57.8%) disagreed with the notion that the adoption of artificial intelligence in the Nigerian healthcare sector could result in job losses. Two-thirds of the participants (66.7%) were of the view that the integration of artificial intelligence in healthcare will augment human intelligence. Three-quarters (77%) of the respondents agreed that the use of machine learning in Nigerian healthcare could facilitate efficient service delivery.

**Conclusion:**

This study provides novel insights regarding healthcare professionals' knowledge and perception with respect to the application of artificial intelligence and machine learning in healthcare. The emergent findings from this study can guide government and policymakers in decision-making as regards deployment of artificial intelligence and machine learning for healthcare delivery.

## Background

Artificial intelligence refers to the various processes involved in the programming of machines that enable them to act like humans and mimic their actions. These activities may include carrying out tasks, processing information, and making decisions based on past events (Mahomed, 2018). According to technological advancements, artificial intelligence may be classified into various systems, including artificial narrow intelligence and artificial general intelligence. Artificial narrow intelligence also called “weak” artificial intelligence, refers to systems that perform a single unique task with human-like proficiency, they have a limited range of capabilities (Harwood et al., 2019). Artificial general intelligence systems have abilities to learn and understand, respond to human emotions, as well as perform multiple tasks like humans (Wahl et al., 2018).

Machine learning is a subset of artificial intelligence which utilizes a set of algorithms and statistical models to enhance computers' abilities to execute relevant tasks (Sanchez and Peters, 2023). The process by which computers are trained to identify patterns in datasets is known as the machine learning method and is divided into several broad classifications, including supervised learning, unsupervised learning, and reinforcement learning (Jovel and Greiner, 2021). The process of supervised learning entails labeling the data used to train the algorithms with the proper answers, if the actual results differ from this, the algorithm learns from it and improves its efficiency (Manne and Kantheti, 2021). In an unsupervised learning model, the algorithm learns from datasets with no predefined output labels and identifies common features such as patterns and relationships in the data, which it then applies to subsequent data (Rong et al., 2020). Reinforcement learning allows the algorithm to receive rewards or punishments for its outputs and promotes the maximization of its rewards over time (Matsuo et al., 2022).

Machine learning can also be variations or combinations of the aforementioned types. Instances include semi-supervised learning (Van Engelen and Hoos, 2020), transfer learning (Weiss et al., 2016), and deep learning. Deep learning uses artificial neural networks with multiple layers to process data (LeCun et al., 2015). Deep learning is a type of machine learning known for its exceptional performance capabilities and its ability to handle complex tasks (Gheisari et al., 2023). This algorithm is able to autonomously identify intricate patterns within large datasets, often requiring minimal human intervention or expertise. Given the need for a large amount of data for deep learning models, sometimes, a trained model from a different but related machine learning application may be used as a starting point (Yu et al., 2022). This approach reduces the need for extensive new datasets and the technique is commonly referred to as “transfer learning.”

The choice of algorithm depends on the nature of the problem and the data at hand, as each form of the algorithm has distinct strengths and drawbacks of its own. Machine learning models may be utilized clinically as a risk assessment tool to predict the chances of developing specific diseases or conditions based on individual patient demographics, medical history, genetic information, and lifestyle factors. This will promote early detection as well as enable early intervention and targeted preventive measures. Furthermore, artificial intelligence may be applied in medical diagnosis, as demonstrated in a study by Ghaderzadeh and Aria (2021). It was reported that artificial intelligence algorithms were incorporated into the diagnosis of COVID-19, resulting in higher degrees of sensitivity and specificity when compared to the traditional Polymerase Chain Reaction test. AI-aided diagnostic techniques can also facilitate the detection and diagnosis of non-communicable diseases, including those with significant public health implications like coronary artery disease (Garavand et al., 2023). In personalized medicine, genetic materials consist of protein sequences, and the study of such sequences holds significance in the identification of genetic markers associated with various diseases. Consequently, this enables the development of drugs and therapeutic agents capable of interacting with these protein sequences to correct a pathological state in the body. Deep learning algorithms are increasingly applied in the task of aligning multiple protein sequences, thereby facilitating the precise and comprehensive identification of similarities and differences within such structures (Fukuda and Tomii, 2020; Baek and Baker, 2022).

Other common artificial intelligence technologies of interest are natural language processing, image and signal processing, expert systems, and robotics. Natural language processing aims to confer the ability to understand human language on computers so as to enable the extraction of information and patterns directly from patients' verbal communication (Park et al., 2020). Image and signal processing artificial intelligence collects and processes data to significantly improve accuracy in diagnoses (Rajeswari and Jagannath, 2017). Some datasets where these have been successfully applied include audio recordings, micrographs, images from medical scans, cries of babies and breath sounds (Ting et al., 2017). Expert systems utilize high-level proficiencies to give advice and proffer solutions regarding the diagnosis and prognosis of medical conditions, as well as educate healthcare professionals (Lee and Wang, 2011). The field of robotics started as an advancement in the field of mechanical and electronic engineering to perform repetitive tasks in industrial settings (Rajan and Saffiotti, 2017). However, the integration of artificial intelligence into such systems has led to the development of intelligent robots that employ a high degree of accuracy and precision to perform complex sophisticated procedures, including surgeries (Bramhe and Pathak, 2022; Alafaleq, 2023). The potential for artificial intelligence in healthcare is numerous, given that it can also be applied in areas such as drug discovery and development, innovative treatment design, and the identification of drug therapy problems (Holzinger et al., 2019).

Studies have highlighted different health applications of artificial intelligence in low and middle-income countries. Examples of these are the use of natural language processing models in epidemiological surveillance and disease prediction, as well as the combination of natural language processing models with expert systems to aid physicians in medical diagnosis (Wahl et al., 2018). Also, artificial intelligence-based chatbots and telehealth platforms have been proposed to address the shortage of health professionals in Africa (Owoyemi et al., 2020). Considering the high malaria burden in Africa and the associated high child mortality rate (World Health Organization, 2023), a new deep-learning model was recently studied to explore its utility in expediting malaria diagnosis and promoting measures to control the disease in Africa. The emergent results from the study indicate significant potential (Oyewola et al., 2022).

Identification and prevention of adverse drug reactions through pharmacovigilance are critical to pharmaceutical care and optimal health outcomes. In a bid to strengthen this process, Oyewusi et al. (2021) assessed a framework that uses natural language processing models to identify and extract cases of adverse drug reactions from open health discussions on social media and other online media conversations. Innovations such as this may be integrated into multidisciplinary efforts aimed at mitigating the negative impacts of adverse drug reactions. Other studies have assessed the potential applications of artificial intelligence in Africa including its use in health insurance plans (Owoyemi et al., 2023), diagnosis of colorectal cancer (Waljee et al., 2022), and the process of drug discovery and development (Patel and Shah, 2022).

Despite international evidence highlighting the prospects of artificial intelligence and machine learning in developing countries' healthcare, there appears to be a paucity of information on the knowledge and perception of healthcare providers regarding the utility of artificial intelligence and machine learning in Nigeria. This study therefore aimed at assessing health professionals' knowledge of artificial intelligence innovations, understanding their perceptions of its prospects, and evaluating their degree of preparedness for its integration into the sector. The findings from this study can help governments and policymakers in developing contextual strategies to guide the adoption of artificial intelligence and machine learning for healthcare services in Nigeria and other low- and middle-income countries.

## Methods

The study was undertaken from March to August 2023. A cross-sectional study design was adopted for the study and responses were obtained from healthcare professionals including physicians, pharmacists, nurses, and medical laboratory scientists.

The questionnaire was designed in the English language following review of the literature (Tran et al., 2019; Siddique and Chow, 2021; van der Schaar et al., 2021; Koçak et al., 2023). The study tool was validated by an expert panel comprising five researchers in this field, following which face and content validations of the data collection instrument were undertaken. Pilot testing was carried out by the administration of the questionnaire to an initial cohort of 20 participants that were randomly selected, the feedback received yielded no major change to the research instrument. The questionnaire contained a demography section and questions structured to gain in-depth understanding of the knowledge and perceptions of respondents regarding artificial intelligence and machine learning applications in healthcare.

A sample size of 384 was calculated for a population of 0.94 million healthcare workers in Nigeria (Ahmat et al., 2022). This was computed at 95% confidence level, 5% margin of error, and 50% response distribution using the Epi Info software version 7. The sample size was rounded up to 500 so as to account for non-response. Data were collected through a stratified multistage sampling technique. Firstly, one state was selected randomly from the six different geopolitical zones in Nigeria. Two health facilities were randomly selected from each state. In each facility, a number of participants were recruited using a convenience sampling technique. The participants were recruited from both public and private healthcare facilities.

Data collected were coded into the Statistical Package for Social Sciences software version 25 and then subjected to relevant analyses. Knowledge of the participants was assessed by thirteen (13) statements with “true,” “false,” and “not sure” answers. The responses were then scored and analyzed using Bloom's cut-off point. Each correct answer was assigned one (1) point whilst incorrect answers and unfilled questions were scored zero (0), the sum of the total score by each respondent was then generated and categorized using Bloom's cut-off point. The overall knowledge was classified as good if the score ranged from 80 to 100% of the total correct answers, moderate if it was between 60 and 79%, and poor if it was below 60%. Inferential analysis was then undertaken using the independent *t*-test and analysis of variance to determine the association between the mean knowledge scores and demographic characteristics. A *post hoc* analysis (Least Significant Difference) was carried out for multiple comparisons in cases of analysis of variance that were statistically significant. A *p*-value < 0.05 was considered significant for the analyses.

Perceptions of the participants regarding the use of artificial intelligence in healthcare were assessed with a total of sixteen statements that utilized a five-point Likert scale to capture participants' views. Univariate analysis was carried out to yield descriptive statistics and data were presented in frequencies and percentages.

## Results

### Demography

A total of 500 questionnaires were administered to participants and only 404 were completed and returned, giving a response rate of 80.8%. The study recorded more responses from female participants (55.7%) compared to their male counterparts (44.3%). Two-thirds of the participants were within the age range of 18 to 30 years (68.3%), whilst respondents above 60 years of age (1.2%) constituted the least proportion. Pharmacists accounted for 27.7% of the participants, and this was closely followed by physicians (24.5%) and nurses (19.3%). Further details about socio-demographic characteristics are presented in Table 1.

**Table 1:** Socio-demographic characteristics of the respondents (*n* = 404).

**Variable**	**Frequency (%)**
**Gender**	
Male	179 (44.3)
Female	225 (55.7)
**Age**	
18–30	276 (68.3)
31–40	84 (20.8)
41–50	26 (6.4)
51–60	13 (3.2)
Above 60	5 (1.2)
**Profession**	
Physician	99 (24.5)
Pharmacist	112 (27.7)
Nurse	78 (19.3)
Medical lab scientist	37 (9.2)
Physiotherapist	26 (6.4)
Others	52 (12.9)
**Highest educational qualification**	
Diploma	23 (5.7)
First degree	332 (82.8)
Master's degree	41 (10.2)
Doctorate	5 (1.2)
**Years of practice**	
< 5 years	285 (71.3)
5–10 years	61 (15.3)
11–15 years	25 (6.3)
Above 15 years	29 (7.2)
**Sector**	
Government sector	279 (71.7)
Private sector	102 (26.2)
Others	8 (2.1)

### Knowledge of healthcare professionals regarding artificial intelligence and machine learning applications in healthcare

The maximum obtainable knowledge score was 13. The average mean knowledge score was 10.37 ± 2.10. Based on Bloom's cut-off point, more than half of the respondents (57.2%) had a good score, about a third of the participants (32.2%) reported average knowledge, and 10.6% of the sample had poor knowledge.

More than half of the participants had correct responses to all the statements except the third statement “machine learning is another name for artificial intelligence,” which had a relatively low frequency of correct responses (24.9%). The first statement “artificial intelligence enables machines to carry out specific tasks with high intelligence like humans” had the highest frequency (96.7%). Other details about the frequency of correct responses are presented in Table 2.

**Table 2:** Frequency of correct responses.

**S/N**	**Statement**	**Frequency (%)**
1.	Artificial intelligence enables machines to carry out specific tasks with high intelligence like a human.	385 (96.7)
2.	Artificial intelligence technologies use information from past events to make decisions.	324 (80.8)
3.	Machine learning is another name for artificial intelligence	100 (24.9)
4.	Machine learning gains understanding from identified patterns in data sets.	349 (88.6)
5.	Image and signal processing may be used to extract information from medical imaging.	353 (88.5)
6.	Machine learning is a subset of artificial intelligence.	300 (75.8)
7.	The use of robotics in surgery is an example of artificial intelligence.	354 (88.3)
8.	Machine learning technologies rely on datasets.	350 (87.7)
9.	Artificial intelligence systems can be used to store health information.	371 (93.5)
10.	Artificial intelligence technologies can improve accuracy in disease diagnosis.	325 (81.3)
11.	Artificial intelligence can be employed in drug information systems.	351 (89.1)
12.	The use of artificial intelligence and machine learning in drug discovery can facilitate the screening of thousands of compounds in a matter of days.	352 (88.0)
13.	The application of artificial intelligence and machine learning techniques in drug discovery is cost-effective compared to traditional methods.	277 (68.7)

### Perceptions of health professionals toward artificial intelligence and machine learning applications in healthcare

**Figure 1:**
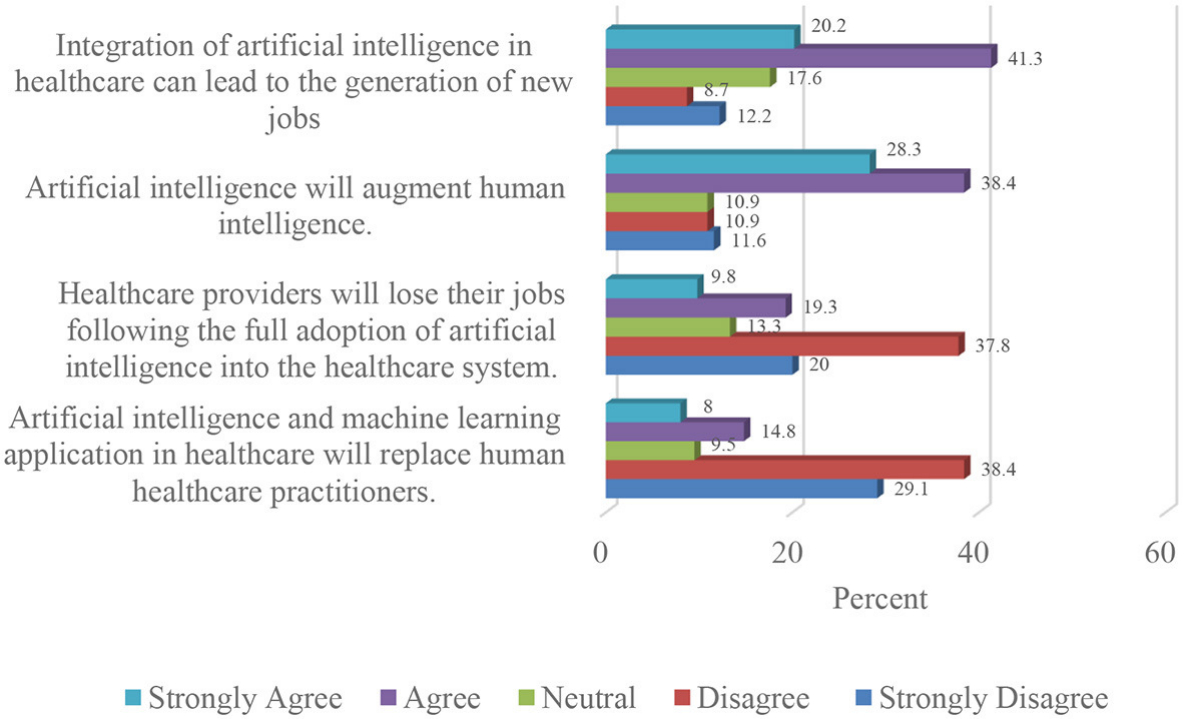
Impact of artificial intelligence and machine learning adoption on the Nigerian healthcare workforce.

From the findings presented in Figure 1, more than half of the respondents (57.8%) disagreed that the adoption of artificial intelligence in the Nigerian healthcare sector would result in job loss. About two-thirds (67.5%) of the participants disagreed that artificial intelligence and machine learning will replace healthcare providers.

The majority of the participants (66.7%) were of the view that the integration of artificial intelligence in healthcare will augment human intelligence. A similar proportion (61.5%) were also of the opinion that integration of artificial intelligence in Nigerian healthcare will result in the generation of new jobs.

Three-quarters (77%) of the respondents agreed that the use of machine learning in Nigerian healthcare will facilitate efficient service delivery. Close to half (48.3%) of the participants disagreed that the adoption of machine learning in the Nigerian healthcare system could increase the incidents of medical errors.

**Figure 2:**
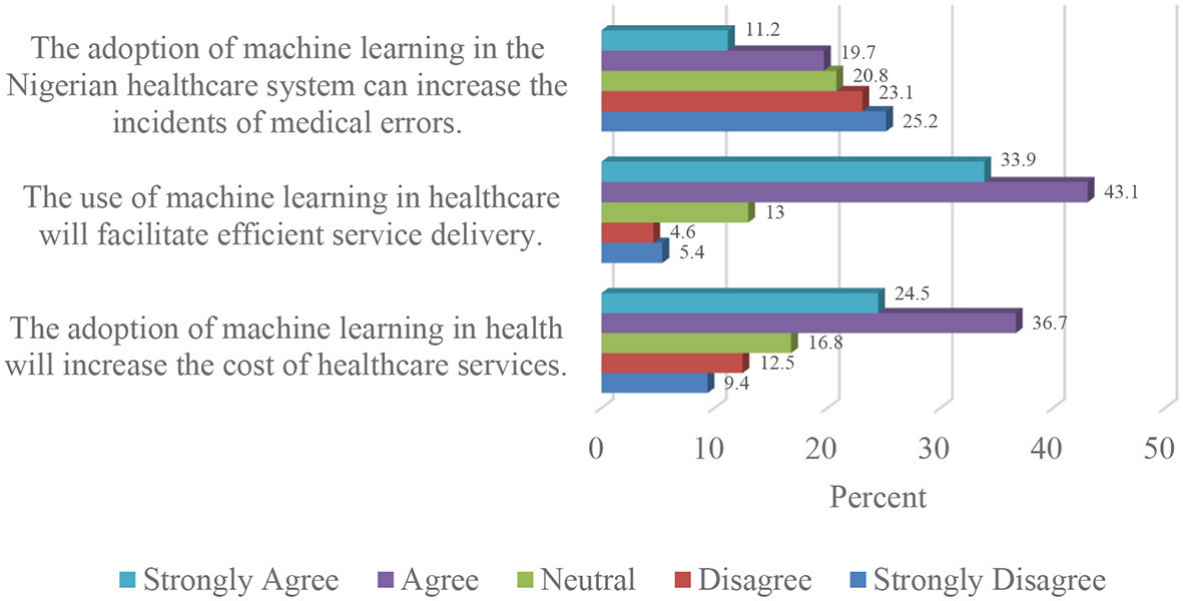
Effect of artificial intelligence and machine learning adoption on Nigerian healthcare delivery.

From Figure 2, almost two-thirds (61.2%) of the study sample opined that machine learning-based health services would be more expensive than traditional healthcare.

Regarding the potential applications of artificial intelligence in Nigerian healthcare, more than two-thirds (68.1%) of the participants agreed that artificial intelligence is essential for healthcare services. Also, 71.9% of the participants believed that artificial intelligence has the potential to enhance the health outcomes of the citizens.

**Figure 3:**
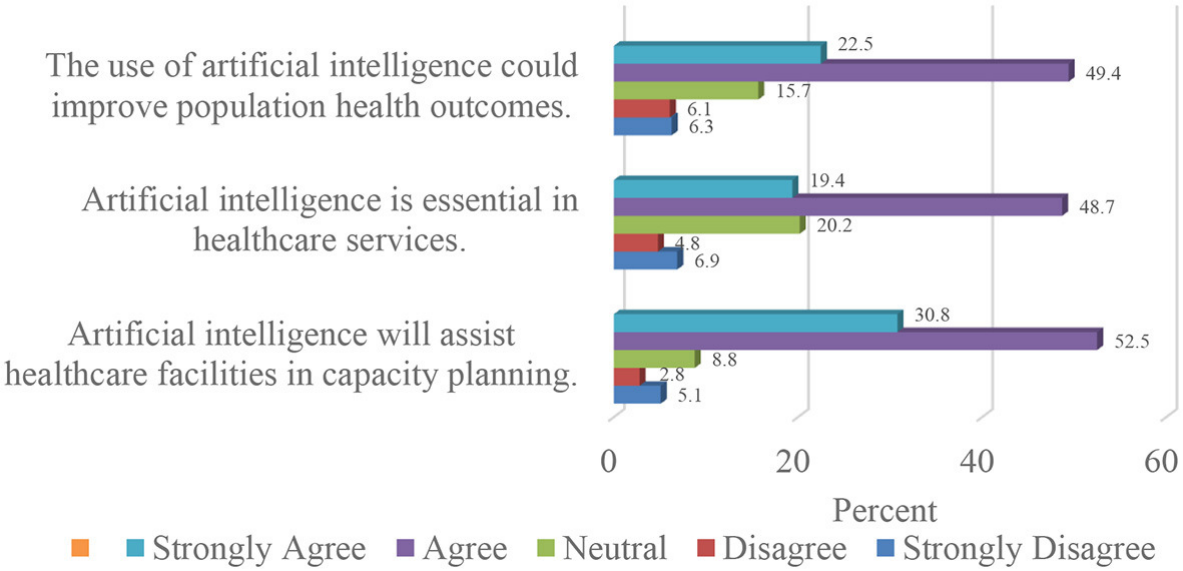
Applications of artificial intelligence and machine learning in Nigerian healthcare.

Furthermore, as presented in Figure 3, more than three-quarters (83.3%) of the respondents were of the opinion that artificial intelligence would aid healthcare facilities in capacity planning.

A strong majority (80.8%) of the respondents were of the view that artificial intelligence in Nigerian healthcare can play an integral role in the sector. In the same vein, three-quarters (74.2%) of the study sample agreed that artificial intelligence can facilitate quality improvement in relevant aspects of healthcare delivery.

**Figure 4:**
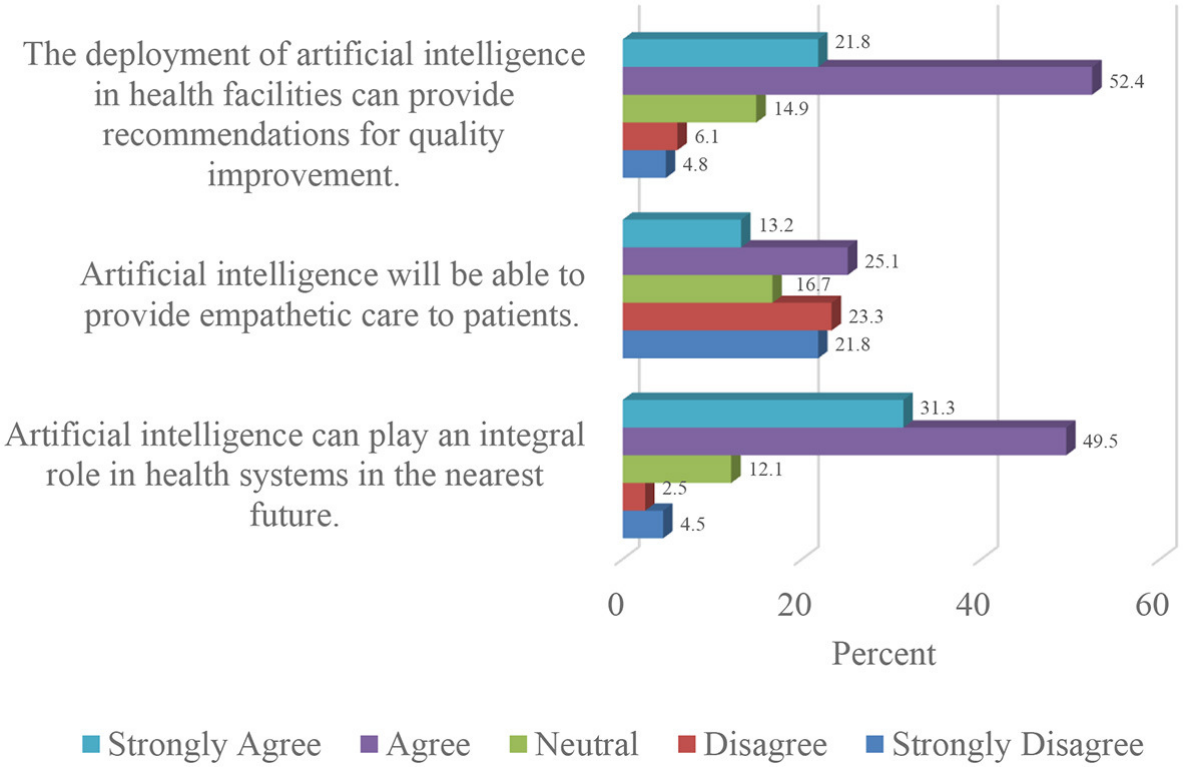
Expectations regarding the adoption of artificial intelligence in Nigerian healthcare.

Conversely, close to half of the participants (45.1%) in the study expressed disagreement regarding the potential of artificial intelligence developing to the stage where it can offer empathetic care to patients. Figure 4 illustrates the outcomes of the respondents' expectations concerning the integration of artificial intelligence in the Nigerian healthcare sector.

**Figure 5:**
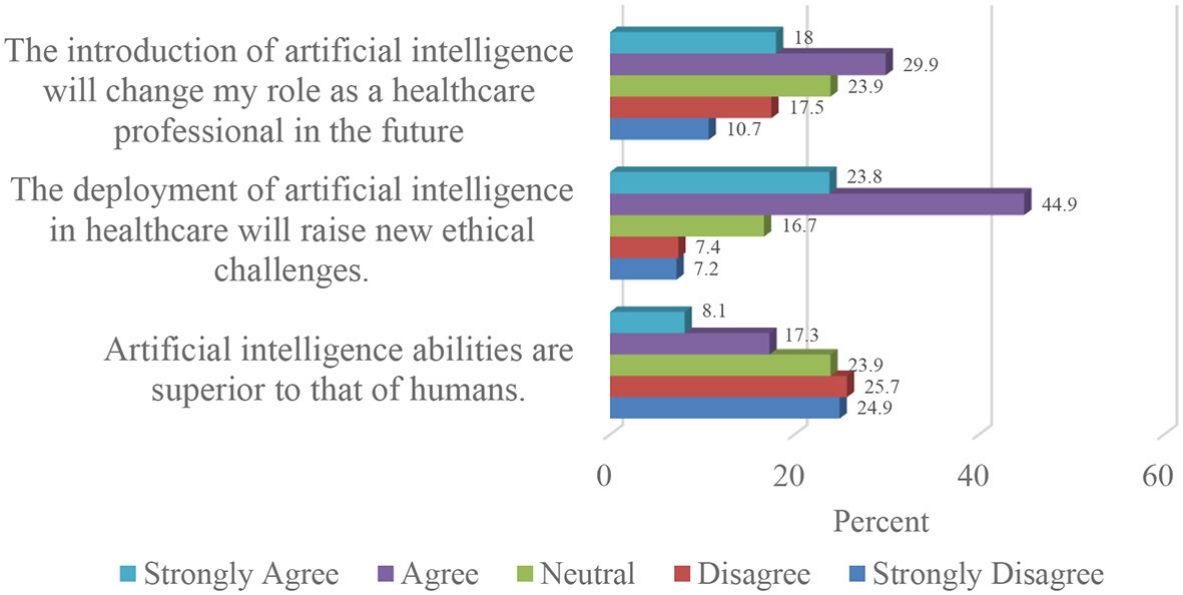
Concerns about the adoption of artificial intelligence in Nigerian healthcare.

In relation to the concerns expressed by respondents regarding the deployment of artificial intelligence in Nigerian healthcare, two-thirds (68.7%) of the study population opined that this would give rise to new ethical challenges. Details are provided in Figure 5.

More than a third (47.9%) of the study sample believed that artificial intelligence would change their roles as healthcare professionals, whilst half (50.6%) of the respondents disagreed that artificial intelligence was superior to human intelligence.

### Association between socio-demographic characteristics and variables

Bivariate analysis revealed a significant relationship between the demographic data and the mean knowledge scores of the participants. The male category had a higher mean knowledge score than the female category (*p-*value = 0.008). A statistically significant difference was also recorded in the category exploring knowledge scores according to professional background (*p-*value = 0.022). The result of the analysis is presented in Table 3.

**Table 3:** Relationship between the knowledge score and socio-demographic characteristics.

**Variable**	**Category**	**Mean ±SD**	**Test of significance (*p*)**
Gender			*t* = 2.652 (0.008)
Male		10.68 ± 1.86	
Female		10.13 ± 2.24	
Age			*F* = 2.258 (0.062)
18–30		10.54 ± 2.04	
31–40		10.17 ± 2.20	
41–50		9.54 ± 2.14	
51–60		10.31 ± 1.89	
Above 60		9.00 ± 2.65	
Profession			*F* = 2.676 (0.022)
Physician	a	10.90 ± 1.87	a vs. c (0.025); a vs. d (0.03); a vs. e (0.003); a vs. f (0.024); b vs. e (0.039)
Pharmacist	b	10.47 ± 2.11	
Nurse	c	10.19 ± 2.11	
Medical lab scientist	d	10.03 ± 2.24	
Physiotherapist	e	9.54 ± 2.45	
Others	f	10.10 ± 2.01	
Highest educational qualification			*F* = 0.064 (0.979)
Diploma		10.43 ± 2.13	
First degree		10.39 ± 2.13	
Master's degree		10.24 ± 1.93	
Doctorate		10.40 ± 1.52	
Years of practice			*F* = 2.470 (0.062)
< 5 years		10.49 ± 2.04	
5–10 years		10.13 ± 2.39	
11–15 years		10.64 ± 1.98	
Above 15 years		9.48 ± 1.98	
Sector			*F* = 0.749 (0.473)
Government sector		10.31 ± 2.16	
Private sector		10.61 ± 1.90	
Others		10.38 ± 2.07	

F, analysis of variance test; t, independent t-test.

## Discussion

Findings from this study revealed that more than half of the respondents had a good knowledge of artificial intelligence and machine learning, alongside their applications in healthcare. The statement about machine learning being another name for artificial intelligence received the least number of correct responses. Artificial intelligence and machine learning are often used interchangeably. However, artificial intelligence is an umbrella term for the development of intelligent machines, whilst machine learning is a subset of artificial intelligence that focuses on creating algorithms that enable machines to learn from data and make predictions (Amaro Junior, 2022). These findings suggest that the participants had a basic knowledge of artificial intelligence, but they lacked a strong grasp of its technical aspects. Efforts to educate health providers regarding artificial intelligence may be targeted at a thorough classification of its subsets and their peculiarities.

Inferential statistics comparing the mean knowledge scores with socio-demographic characteristics revealed that physicians and pharmacists had a higher mean score than other healthcare professionals. Physicians are directly involved in clinical decision-making; this exposes them to complex medical cases requiring accurate diagnoses and formulation of evidence-based treatment plans. Artificial intelligence technologies, including machine learning algorithms, are increasingly being used in that regard hence physicians who actively use artificial intelligence tools in their clinical practice will likely have a deeper understanding of artificial intelligence (Basu et al., 2020). Similarly, a higher mean knowledge score by pharmacists could be due to a progressive increase in the use of artificial intelligence innovations for drug design and development in the pharmaceutical sector (Raza et al., 2022; Stasevych and Zvarych, 2023). These novel findings clearly identify potential early adopters of the technology, and this can be useful in policy articulation and implementation planning. Although, this study reported a higher knowledge score amongst physicians and pharmacists, interdisciplinary collaboration among various health professionals is essential for successful artificial intelligence implementation in healthcare. Hence, more research targeted at identifying factors contributing to knowledge disparities between the different groups of health professionals is of key importance.

Male respondents had a higher mean knowledge score than their female counterparts and this was statistically significant. One possible explanation for this phenomenon may be perhaps due to the higher interest usually expressed by males toward the use of information and communication technologies (Cai et al., 2017).

In this study, half of the respondents were confident that the adoption of artificial intelligence in healthcare will augment human intelligence and generate new jobs as opposed to the popular belief that its use can lead to job loss (Butcher and Beridze, 2019; Abdullah and Fakieh, 2020). This is similar to a study undertaken in Korea (Oh et al., 2019). Similarly, the respondents disagreed that artificial intelligence will replace human healthcare professionals. A possible rationale for these findings could be the historical precedence of technological advancements leading to the creation of new jobs. Hence, some of the health professionals believed that artificial intelligence technologies will likely follow a similar pattern and create new jobs. Artificial intelligence can enhance clinical decision-making and enable innovative solutions across other areas of healthcare, this can lead to the emergence of new job opportunities to support and advance artificial intelligence-based innovations (Tursunbayeva and Renkema, 2022).

Close to half of the participants in this study disagreed that artificial intelligence can increase the incidents of medical errors and more than half were of the opinion that artificial intelligence can facilitate efficient service delivery in healthcare. Furthermore, a significant proportion of the study sample believed that the integration of machine learning in the Nigerian health sector can increase the cost of health services, and this is similar to earlier findings by Richardson et al. (2021) where similar views were reported by patients. This is a serious concern because payment for health services in Nigeria is majorly through out-of-pocket (Ephraim-Emmanuel et al., 2018). Hence, this concern has the potential of stalling the acceptance of artificial intelligence by the public and it may further limit access to healthcare. One potential solution to prevent a high cost for artificial intelligence-based health services is to promote collaborative efforts between the government, private sector, developmental partners, and other donors to create funding mechanisms for artificial intelligence adoption in healthcare (Reddy et al., 2019). Furthermore, contextual cost-effectiveness studies aimed at assessing the overall benefits of artificial intelligence and its potential to optimize resource utilization and enhance productivity are important to assure the public of the criticality of artificial intelligence in healthcare (Gomez-Rossi et al., 2022).

The findings from this study indicate that a significant proportion of the participants recognized the significance of artificial intelligence in various aspects of healthcare. The majority of the study participants agreed that artificial intelligence was essential for high-quality health services; good population health outcomes; healthcare facilities' capacity planning; and quality improvements. These are consistent with a previous study undertaken by Robinson (2020), which focused on two teaching hospitals in the southern part of Nigeria. This reflects a growing understanding of artificial intelligence capabilities to transform and optimize healthcare delivery. Through the use of cutting-edge technologies and data-driven algorithms, artificial intelligence has the potential to enhance diagnostic accuracy, streamline healthcare workflows, enable personalized treatment approaches, and improve overall patient outcomes (Matsuo et al., 2022). This underscores the importance of further exploration of artificial intelligence technologies in the healthcare sector to unlock its full potential and advance the delivery of healthcare services in Nigeria.

The study revealed that more than a third of the participants disagreed that artificial intelligence systems can provide empathetic care whilst a similar fraction of the study sample believed that artificial intelligence can change their roles as healthcare professionals. The ability to effectively understand and respond to the emotional and psychological needs of patients are critical elements in providing compassionate and empathetic healthcare. Artificial intelligence currently lacks the capability to provide empathetic care, however, its utilization in healthcare can enhance efficiency and provide healthcare professionals with additional free time (Kerasidou, 2020). Approximately half of the respondents disagreed that artificial intelligence is superior to human intelligence. This disagreement highlights the belief that human intelligence is associated with unique qualities and capabilities that cannot be replicated or surpassed by artificial intelligence technologies (Morrow et al., 2023).

Despite agreeing that artificial intelligence would play an integral role in future healthcare, two-thirds of the respondents were of the view that the adoption of artificial intelligence can raise new ethical challenges. This is similar to the report from a study in China (Wu et al., 2023). Patients' confidentiality is essential for high-quality healthcare delivery. Patients' data are legally protected and should be secured against unauthorized access (Karasneh et al., 2021). However, large datasets are needed for training artificial intelligence models, hence, concerns about privacy and safety can arise when medical data are collected and shared (Esmaeilzadeh, 2020). This also raises the issue of algorithmic bias because machine learning can only produce results that are as accurate as the data they are trained on, and if this data is not representative of the entire population the program is designed to serve, then the results may be discriminatory (Panch et al., 2018). To overcome these challenges, data governance frameworks, ethical guidelines, and supportive policies are essential. Collaboration amongst stakeholders and contextual knowledge-sharing policies can play a key role in harnessing the potential of artificial intelligence and improving healthcare delivery in Nigeria.

## Conclusion

This study provides novel insights into the perspectives of healthcare professionals in Nigeria regarding artificial intelligence integration in the health sector. It emerged that a significant proportion of the study population had good knowledge of artificial intelligence. There is increasing acceptance of artificial intelligence worldwide in various sectors, hence health providers are encouraged to inculcate basic knowledge of artificial intelligence and its applications in continuing professional development. More than half of the respondents believed that artificial intelligence would complement human efforts rather than replace it. They were also of the opinion that artificial intelligence could revolutionize healthcare.

Based on the findings of this study, Nigerian health professionals exhibited a high level of readiness and enthusiasm for the adoption and implementation of artificial intelligence innovations. The *status quo* suggests the possibility of expeditiously developing emergent policies, frameworks and collaborative models can leverage artificial intelligence to improve effectiveness and efficiencies of existing systems, as well as the realization of new prospects. It is however imperative to undertake further research to deepen some of these emergent findings that relate to artificial intelligence in the Nigerian healthcare system. This would exponentially improve its implementation and consequent impact in the sector. It will also enable rapid identification of potential hindrances to the effective adoption of artificial intelligence technologies in the Nigerian setting, as well as signpost contextual mitigatory measures to address them.

## Data availability statement

The raw data supporting the conclusions of this article will be made available by the authors, without undue reservation.

## Ethics statement

This study was approved by National Institute for Pharmaceutical Research and Development Health Research Ethics Committee. The research was conducted in accordance with the local legislation and institutional requirements. The participants provided written informed consent to participate in this study.

## Author contributions

OA: Conceptualization, Data curation, Formal analysis, Investigation, Methodology, Supervision, Validation, Writing—review & editing. GO: Data curation, Formal analysis, Investigation, Methodology, Project administration, Validation, Writing—original draft. SS: Data curation, Formal analysis, Investigation, Methodology, Validation, Writing—original draft.
