# Supplementary material for: Exploring the matrix: knowledge, perceptions and prospects of artificial intelligence and machine learning in Nigerian healthcare
Source: Front Artif Intell. 2024 Jan 19;6:1293297. doi: 10.3389/frai.2023.1293297 (PMC10834749; doi:10.3389/frai.2023.1293297)
Supplement: Supplementary file 1 [file Table_1.DOCX]

**Questionnaire**

**Exploring the Matrix: Knowledge, Perceptions and Prospects of Artificial Intelligence and Machine Learning in Nigerian Healthcare**

**Introduction**

Artificial Intelligence (AI) refers to the simulation of human intelligence in machines that are programmed to act like humans and mimic their actions. This questionnaire aims to assess the knowledge and perception of healthcare professionals regarding the applications of AI in healthcare. Please fill out the questionnaire by ticking (√) the most appropriate option (s). Your responses will be anonymised and treated confidentially.

**Demographic Data**

1. **Gender**

| Male | Female |
| --- | --- |

1. **Age**

| 18-30 | 31-40 | 41-50 | 51-60 | Above 60 |
| --- | --- | --- | --- | --- |

1. **Profession**

| Physician | Pharmacist | Nurse | Medical Laboratory Scientists | Physiotherapist | Others, please specify  ……………….. |
| --- | --- | --- | --- | --- | --- |

1. **Highest Educational Qualification**

| Diploma | First degree | Master’s degree | Doctorate |
| --- | --- | --- | --- |

1. **Number of Years of Practice**

| >5years | 5-10years | 11-15years | Above 15years |
| --- | --- | --- | --- |

1. **Sector**

| Government Sector | Private Sector | Others, please specify ………………………………. |
| --- | --- | --- |

1. **Knowledge of Artificial Intelligence and Machine Learning**

| **SN** | **Statement** | **True** | **False** | **Not Sure** |
| --- | --- | --- | --- | --- |
|  | Artificial intelligence enables machines to carry out specific tasks with high intelligence like a human. |  |  |  |
|  | Artificial intelligence technologies use information from past events to make decisions. |  |  |  |
|  | Machine learning is another name for artificial intelligence |  |  |  |
|  | Machine learning gain understanding from identified patterns in data sets. |  |  |  |
|  | Image and signal processing may be used to extract information from medical imaging. |  |  |  |
|  | Machine learning is a subset of artificial intelligence. |  |  |  |
|  | The use of robotics in surgery is an example of artificial intelligence. |  |  |  |
|  | Machine learning technologies rely on datasets. |  |  |  |
|  | Artificial intelligence systems can be used to store health information. |  |  |  |
|  | Artificial intelligence technologies can improve accuracy in disease diagnosis. |  |  |  |
|  | Artificial intelligence can be employed in drug information systems. |  |  |  |
|  | The use of artificial intelligence and machine learning in drug discovery can facilitate the screening of thousands of compounds in a matter of days. |  |  |  |
|  | The application of artificial intelligence and machine learning techniques in drug discovery is cost-effective compared to traditional methods. |  |  |  |

**Perceptions of Health Professionals regarding Artificial Intelligence and Machine Learning Applications in Healthcare**

1. **Impact of Artificial Intelligence and Machine Learning Adoption on the Nigerian Healthcare Workforce**

| **SN** | **Statements** | **Strongly Disagree** | **Disagree** | **Neutral** | **Agree** | **Strongly Agree** |
| --- | --- | --- | --- | --- | --- | --- |
|  | Artificial intelligence and machine learning application in healthcare will replace human healthcare practitioners. |  |  |  |  |  |
|  | Healthcare providers will lose their jobs following the full adoption of artificial intelligence into the healthcare system. |  |  |  |  |  |
|  | Artificial intelligence will augment human intelligence. |  |  |  |  |  |
|  | Integration of artificial intelligence in healthcare can lead to the generation of new jobs |  |  |  |  |  |

1. **Effect of Artificial Intelligence and Machine Learning Adoption on Nigerian Healthcare Delivery.**

| **SN** | **Statements** | **Strongly Disagree** | **Disagree** | **Neutral** | **Agree** | **Strongly Agree** |
| --- | --- | --- | --- | --- | --- | --- |
|  | The adoption of machine learning in health will increase the cost of healthcare services. |  |  |  |  |  |
|  | The use of machine learning in healthcare will facilitate efficient service delivery. |  |  |  |  |  |
|  | The adoption of machine learning in the Nigerian healthcare system can increase the incidents of medical errors. |  |  |  |  |  |

1. **Applications of Artificial Intelligence and Machine Learning in Nigerian Healthcare**

| **SN** | **Statements** | **Strongly Disagree** | **Disagree** | **Neutral** | **Agree** | **Strongly Agree** |
| --- | --- | --- | --- | --- | --- | --- |
|  | Artificial intelligence will assist healthcare facilities in capacity planning. |  |  |  |  |  |
|  | Artificial intelligence is essential in healthcare services. |  |  |  |  |  |
|  | The use of artificial intelligence could improve population health outcomes. |  |  |  |  |  |

1. **Expectations Regarding the Adoption of Artificial Intelligence in Nigerian Healthcare**

| **SN** | **Statements** | **Strongly Disagree** | **Disagree** | **Neutral** | **Agree** | **Strongly Agree** |
| --- | --- | --- | --- | --- | --- | --- |
|  | Artificial intelligence can play an integral role in health systems in the nearest future. |  |  |  |  |  |
|  | Artificial intelligence will be able to provide empathetic care to patients. |  |  |  |  |  |
|  | The deployment of artificial intelligence in health facilities can provide recommendations for quality improvement. |  |  |  |  |  |

1. **Concerns about the Adoption of Artificial Intelligence in Nigerian Healthcare**

| **SN** | **Statements** | **Strongly Disagree** | **Disagree** | **Neutral** | **Agree** | **Strongly Agree** |
| --- | --- | --- | --- | --- | --- | --- |
|  | Artificial intelligence abilities are superior to that of humans. |  |  |  |  |  |
|  | The deployment of artificial intelligence in healthcare will raise new ethical challenges. |  |  |  |  |  |
|  | The introduction of artificial intelligence will change my role as a healthcare professional in the future |  |  |  |  |  |
